# Quality of evidence in a post-Soviet country: evaluation of methodological quality of controlled clinical trials published in national journals from Uzbekistan

**DOI:** 10.1186/s12874-020-01076-x

**Published:** 2020-07-13

**Authors:** Timur Aripov, Dilfuza Aniyozova, Irina Gorbunova

**Affiliations:** 1 Department of Public Health and Healthcare Management, Tashkent Institute of Postgraduate Medical Education, Parkentskaya str. 51, Tashkent, Uzbekistan 100007; 2 Principal investigator at Antimicrobial Resistance Research project, Tashkent Institute of Postgraduate Medical Education, Parkentskaya str. 51, Tashkent, Uzbekistan 100007

**Keywords:** Public health, Evidence-based medicine, Healthcare quality, Randomized controlled trial, Uzbekistan

## Abstract

**Background:**

Most researchers in Uzbekistan prefer to publish their reports in journals of their home country. Moreover, the proportion of healthcare practitioners who prefer to use these national sources of information also remains high. However, the quality of publications from national journals, in post-Soviet countries, has not been systematically evaluated until now. The primary objective of this study was to evaluate the quality of randomized controlled trials’ (RCTs) reports published in medical journals from Uzbekistan. We supposed that reports had at least minimal quality to contribute to the higher quality of healthcare.

**Methods:**

To evaluate the quality of RCTs, we selected two journals from the list of national medical journals for which background information was provided. We decided to select articles from journals that had the highest subscription rate and were likely to have the highest impact on clinical decisions. The journals were *Medical Journal of Uzbekistan* and *Paediatrics*. Only issues published in 2007–2017 were considered for evaluation. Two evaluators independently scored RCTs and controlled clinical trials (CCTs) reported in the journals. The 5-point scale developed by Jadad et al. was used to evaluate the quality of reports. Consensus-based decision was made about the final score of each report.

**Results:**

We reviewed 1311 studies in the two journals and found 380 clinical trials reports for the final evaluation. Our main finding was that none of the reports received a final score of more than 1, with an absolute agreement between evaluators. A median score of the studied reports was equal to 0, predicting a very low quality of controlled trials reported in the national journals (Wilcoxon signed-rank test *p* = 1.0; 95% CI = 0–0).

**Conclusions:**

We believe that quality of reports about controlled trials, in Uzbekistan, can be considered insufficient to contribute to the higher quality of care and patients’ safety. In the worst case, such condition can cause serious damage to the public health and lead to ineffective use of resources in the country. Therefore, the better reporting and organization of RCTs and CCTs should become a main goal of all stakeholders interested in the effective and safe healthcare in the country.

## Background

In post-Soviet countries, language barriers and poor access to international databases in medical schools and facilities have isolated students and practitioners from high-quality evidence. These factors made a contribution to the low quality of healthcare, in those countries [1, 2]. Moreover, such isolation contributed to outdated study designs, with low incentives for researchers to organize scientifically rigorous studies. The limited availability of foreign medical literature slowed down integration into the international scientific community, hindering the advancement in medical researches [3]. A weak attention to the evidence in the framework of under- and postgraduate medical education became an additional impediment to higher quality of studies [4, 5]. As a result, a poor design of studies, combined with an improper statistical analysis, restricted an inclusion of Russian medical publications in meta-analyses round the world [6].

A concept, supported by WHO, emphasizes the growing importance of well-designed primary studies from middle- and low-income countries in improving guidance of health systems [7]. The role of high-quality randomized controlled trials (RCTs) becomes crucial in addressing this concept. Until now, reviews of the quality of reports in post-Soviet countries covered only studies from Russia or close neighbouring countries. In the last period of time, the popularity of international databases such as PubMed increased in all post-Soviet countries, including Uzbekistan [2]. Researchers and medical professionals, in those countries, presumably became less isolated from global science than they were previously. However, the portion of researchers who publish their reports in national journals, which are not indexed in international databases, remains still high [8]. Moreover, the proportion of healthcare practitioners who prefer to use national sources of information also remains high [9]. Despite those facts, the quality of publications from national journals has hardly ever been systematically evaluated. It is known that most journals in the world accept CONSORT checklist as a submission guideline for reported RCTs [10]. However, it is not clear whether such checklists are used in the national journals and how their use impacts on the quality of the reports. As a method for evaluation, Cochrane Risk of Bias (RoB) is a widely preferred tool, this time [11].

The primary objective of this study was to evaluate the quality of RCTs’ reports published in medical journals from Uzbekistan. An original hypothesis supposed that reports had at least minimal quality to contribute to the higher quality of healthcare. We decided to evaluate reports published in national journals during at least one-decade period.

## Methods

For our study, we selected journals from a list published on Uzbek Ministry of Health (MoH)‘s webpage [12]. After the exclusion of newspapers and magazines, nine journals from that list were considered for the study. Due to the budgetary concerns, we were not able to evaluate reports from all nine journals. Because it was technically impossible to identify all RCTs’ reports published in the journals and randomly select from them, we applied a non-random approach. We decided to select articles from journals that had the highest subscription rate and were likely to have the highest impact on clinical decisions made by national practitioners. So, we picked two journals from nine, for which information about their aims and scopes were provided and which had the highest subscription rates. The first journal was *Medical Journal of Uzbekistan*, founded in 1922; the second was *Paediatrics*, founded in 1996. Both journals were registered by the ISSN International Centre and had unique codes. They published reports in different areas of medicine, while the rest of journals covered only specific subjects such as immunology, dermatology, cardiology, neurology and surgery. Either selected journal published articles only in Russian or Uzbek, with providing abstracts in English.

### Inclusion criteria

We analyzed reports published in the two journals during an 11 year period (2007–2017). For criteria to include studies, we referred to Cochrane definitions of RCTs and controlled clinical trials (CCTs) [13]. We evaluated all reports of RCTs and CCTs, in which individuals were “definitely or possibly assigned prospectively to one of two (or more) alternative forms of health care”. An explicit use of some variant of the term “random” in the report classified the trial into RCTs. The rest of the trials were considered CCTs, and because randomization could not be ruled out in them, they were also included to the analysis.

We conducted handsearching in both electronic and paper issues of journals to find all reports of RCTs and CCTs. The search strategy suggested screening the titles and abstracts to spot information related to inclusion criteria. This strategy helped to find studies in which “randomized controlled trial” or “controlled clinical trial” was not mentioned in their titles. Full reports were obtained only for studies where there was insufficient information in the title and abstract. One researcher (IGG) selected reports, and only those approved by other two evaluators were selected for the final evaluation with the use of the assessment tool. Reports written in either Russian or Uzbek were included in the study. We extracted an information about the sample size, types of interventions, outcomes measured and authors involved for all included trials.

### Evaluation tool

The assessment instrument developed by Jadad et al. [14] was used to evaluate the quality of published RCTs and CCTs. Characteristics of this tool are comparable, in terms of accuracy and reliability, to Cochrane RoB tool [15, 16]. The Jadad scale is still used widely for systematic assessments of RCTs, and it does not require the intense training of reviewers, a condition required to provide for high reliability of the RoB method [17–21].

As procedure requires, first, we evaluated each report according to the following three primary questions to detect the sources of the possible bias in RCTs and CCTs:

1. Was the study described as randomized (this concept includes the use of words such as randomly, random, and randomization)?
2. Was the study described as double blind?
3. Was there a description of withdrawals and dropouts?

Each question entailed a “yes” or “no” response option. For every “yes” a score of 1 point and for every “no” 0 points were given. Only whole numbers and no in-between marks were acceptable for the analysis. Each article was assessed and scored independently by two evaluators (TYA and DJA), who also later arrived at a consensus score. In the second step, we evaluated the appropriateness of the randomization and double blinding procedures by additional criteria. By those criteria, scores awarded for the first two questions could be upscored (+ 1 or + 2 points) or downscored (− 1 or − 2 points). The final score that each report received could range from 0 to 5, with a score of 3 or more indicating superior quality [14]. Consensus meetings were conducted in case of a disagreement between evaluators. The third author (IGG) served as the arbiter in the deciding on a consensus score.

### Statistical analysis

Two evaluators independently scored RCTs and CCTs reported in the journals. The decision about the trial score could range on an ordinal scale from 0 to 5. Consensus-based data were collected on an Excel spreadsheet [see Supplementary file 1] and exported to Minitab (v.18, Minitab Inc., USA) for analysis. A statistical (*H*_0_) hypothesis was that RCTs and CCTs reported in journals of Uzbekistan had a median score of equal or less than 1. In a descriptive step, we provided a median score of the evaluated reports. A nonparametric Wilcoxon signed-rank test for a population median was used to test the hypothesis. As an index of the interrater agreement, calculation of Cohen’s unweighted or weighted kappa (*κ*) was accepted appropriate [22–24]. The interpretation of kappa (*κ*) was based on the values given in Table 1.

**Table 1:** Interpretation of the kappa values suggested by Landis and Koch [22]

Kappa (*κ*)	Interpretation
< 0.00	Poor
0.00–0.20	Slight
0.21–0.40	Fair
0.41–0.60	Moderate
0.61–0.80	Substantial
0.81–1.00	Almost perfect

## Results

### Selection of studies

**Fig. 1:**
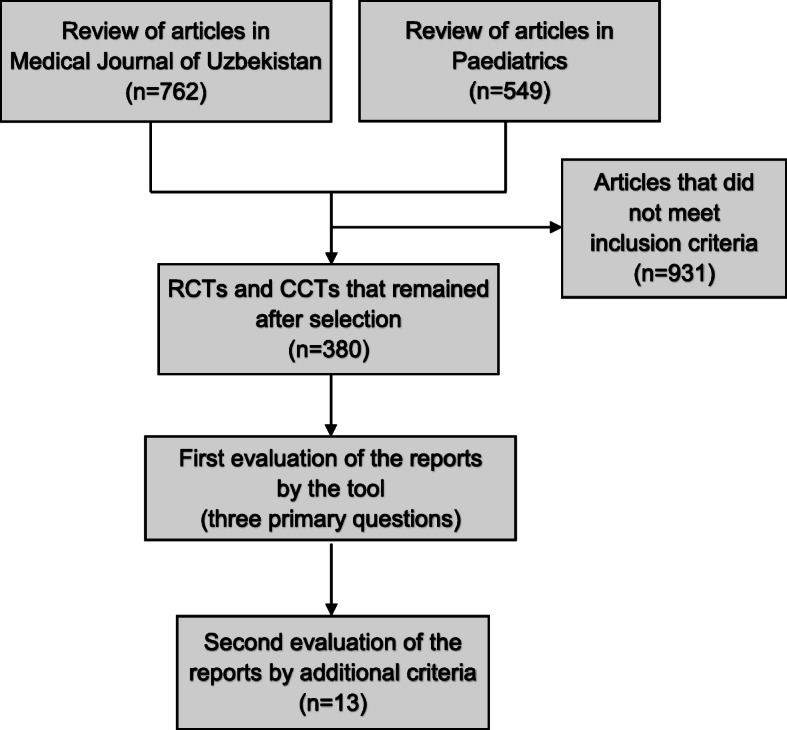
Flow diagram of a review and a study selection process

We reviewed 1311 studies in the two journals to find reports of RCTs and CCTs. The number and proportion of clinical trials varied greatly in issues of the journals. Neither journal had specific requirements for reporting results of RCTs or CCTs and for registration of the trials. One journal required authors to follow the International Committee of Medical Journal Editors (ICMJE) uniform requirements for the publishing reports in medical journals. Because reports of trials could be potentially published in various sections of the journals, we reviewed all sections per issue. We noted that none of the selected trials had mentioned “randomized controlled trial” or “controlled clinical trial” in their title. A review and a study selection process are outlined in Fig. 1.

We included 380 clinical trials’ reports for the final evaluation. The sample size in the included trials ranged from 9 to 2563, and 117 studies had 100 or more participants. The number of groups, their characteristics and the types of interventions in the trials, also varied in the both journals. Only trials reported in *Paediatrics* engaged < 18 years-old participants as a specific group of all cases. In 32% of reports, published in both journals, clinically important outcomes were replaced by unimportant (surrogate) biochemical or other endpoints, supposedly attributed to some diseases or conditions. In 71% of all reports, the trials involved no more than three authors. Statisticians were declared to be involved in 2.9% of all studies, and in none of studies they were involved as coauthors. The categorical description of the included trials is provided in Table 2.

**Table 2:** Categorical description of the trials evaluated in the study (*n* = 380)

Characteristic assessed	Subgroup	Medical Journal of Uzbekistan (*n* = 233) No. (%)	Paediatrics (*n* = 147) No. (%)
Sample size	< 100	164 (70.4)	99 (67.3)
	100–499	61 (26.2)	45 (30.6)
	500–1000	6 (2.6)	2 (1.4)
	> 1000	2 (0.8)	1 (0.7)
Types of interventions	Chemotherapy	157 (67.4)	113 (76.9)
	Surgical	13 (5.6)	6 (4.1)
	Herbal	27 (11.6)	6 (4.1)
	Other	36 (15.4)	22 (14.9)
Outcomes measured	Clinically important	97 (41.6)	93 (63.4)
	Unimportant	94 (40.3)	27 (18.3)
	Mixed	42 (18.1)	27 (18.3)
Authors	< 4	177 (76.0)	93 (63.3)
	4–6	43 (18.4)	33 (22.4)
	> 6	13 (5.6)	21 (14.3)
Statistician involvement	No mention	226 (97.0)	143 (97.3)
	Involvement declared	7 (3.0)	4 (2.7)
	Involved as coauthor	Not identified	Not identified

Most included reports had such special sections as Introduction, Methods and Conclusions, in their structure. We also noticed that none of reports contained special sections to discuss results of the trials and their limitations. Another notable issue was that authors of controlled trials emphasized positive effects of interventions in their study, with minimal discussion of negative outcomes. None of reports contained special notes about the risk of conflict of interest, in authors.

### Main results

The main finding of our analysis was that none of the reports received a final score of more than 1, with an absolute agreement between the two evaluators (TYA and DJA). The proportion of reports that received any score except of 0 was only 4.2 and 2.7% in *Medical Journal of Uzbekistan* and in *Paediatrics,* respectively. The median score of the studied reports was equal to 0, predicting very low quality of controlled trials reported in the national journals (Wilcoxon signed-rank test *p* = 1.0; 95% CI = 0–0).

Intermediate scores were based on the evaluation according to the three primary questions. We considered only specific terms that could be used in the text of the reports. In this step, an agreement rate between the two evaluators was moderate or high. In cases there were initial disagreements, they were resolved and a consensus score was used. The distribution of the consensus-based decisions for all items is provided in Table 3.

**Table 3:** Distribution of consensus-based decisions for all items in the Jadad’s assessment tool (*n* = 380)

Item	n^a^	Adequate (%)	Inadequate (%)	*κ* (95% CI)
Randomly, random or randomization	380	2.9	97.1	0.59 (0.34–0.86)
Double blinding	361	0.6	99.4	0.79 (0.40–1.00)
Withdrawals and dropouts	380	4.5	95.5	0.61 (0.42–0.79)
Appropriateness of the randomization	11	0	100	Agreed absolutely
Appropriateness of the double blinding	2	0	100	Agreed absolutely

^a^ The number is less than 380 for certain items, as there were trials in which certain items were not applicable or not relevant

Additional criteria were used to evaluate the appropriateness of randomization and double blinding, and they were crucial to final scores of the reports. These criteria were applied only to reports that received some points in the initial evaluation. Therefore, 11 reports were applicable to evaluation of the randomization and two reports to evaluation of the double blinding. However, both randomization and double blinding were rated as inappropriate in all included reports, with an absolute agreement between evaluators.

Reports of controlled trials received a final score of 1 in cases where their authors explicitly stated that there was no withdrawals or dropouts of participants from the trials. Both evaluators decided to award a score of no more than 1 and to downscore all reports of the trials in which the randomization and double blinding were declared but were not described in appropriate details.

## Discussion

The results of this study showed a very poor quality of RCTs and CCTs published in the two national journals of Uzbekistan, from 2007 to 2017. Specifically, these findings were related to the reporting of such key components of clinical trials as the random assignment of participants and the double blinding of the interventions. The proportion of the reports, in which specific descriptions were provided, was critically small in both journals. However, those reports were not given a minimal final score because the randomization and double blinding were not reported appropriately.

The quality of reports that we found on our study differs from the global trend that shows an overall increase in the quality of reported trials. Two reviews, evaluating the quality of RCTs, concluded that the poor reporting and the use of inadequate methods had decreased over the last decade, especially regarding sequence generation and allocation concealment [25, 26]. However, in both reviews, the Cochrane RoB tool was used to evaluate quality of the trials. A recent study showed that authors using the RoB tool often make scoring mistakes [27]. In those reviews, a higher journal impact factor was associated with a lower proportion of trials rated as unclear or having a high risk of bias. In this respect, we found that neither of two journals tracked the progress with citations of its articles in any way. We suggest that both journals have a high rate of national citations because of their popularity among researchers. However, as we found, such popularity did not reduce the risk of bias in the trials reported on them.

Our findings raise concern that many clinicians in Uzbekistan still apply low-quality clinical evidences in their practice. This problem can be critical for practitioners who have poor English-language proficiency or find the Internet confusing. The number of such practitioners remains high in Uzbekistan [9]. Lack of skills in the critical assessment of the information aggravates the problem. In this situation, practitioners have no alternative but to use poor evidences from available national journals. Therefore, it is important to improve all language courses, including those in an undergraduate curriculum. Existing courses of English should include skills in the reading of articles from medical journals, the basics of language proficiency in medicine. Regular trainings of clinicians in how to use international databases and to evaluate quality of reports could have an important additional benefit. A study demonstrated that such educational approach would contribute to the use of better evidences in the practice [28].

In addition to the lack of the good evidence for practitioners, the poor quality of trials raises ethical issues related to respecting the rights of patients involved in such studies. A collection of informed consent, in such trials, cannot address its primary mission which is to get feedback from human subjects who can consider all possible risks. It is unrealistic to expect that consent forms informed patients that they would, possibly, take part in the low-quality trial. A personal risk of participating in the poorly designed trial can be high, and such risk hardly can be predicted and accepted by the researcher. From a justice view, a non-random or unclear approach, reported in the national trials, can contribute to unfair allocation of their participants. In this way, researchers can allocate subjects of the study, basing on such unacceptable criteria as income or education level of the subjects or their ability to stand up for their own rights.

If we consider that about a third of the trials evaluated in our study engaged more than 100 participants, the amount of resources spent inefficiently becomes important. In this regard, the formal registration of trials and their ethical assessment, at least at the national level, could play a positive role in reducing publication bias [29–31]. However, neither journal on our study required registration of trials either at the time of a submission or a peer review of reports. The formal instructions for organization of clinical trials in Uzbekistan are provided in a MoH decree, and such issues as the randomization and blinding are highlighted in that decree. A notable finding of our analysis was that none of the evaluated studies were indicated as a “randomized controlled trial” or “controlled clinical trial” in their title.

It becomes clear that journals, we examined, prefer to accept for publication all that is submitted by authors. In practice, every journal should instead require from authors – even if they submit controlled trials not indicated as RCTs – to follow specific rules for the reporting RCTs. Neither of the journals in our study required authors to follow such rules. Both journals were concerned only with the structure of articles and provided guidelines for the text formation. One of the journals instructed authors to follow the ICMJE uniform requirements. But the “preparing for submission” section of those requirements clearly provides CONSORT as the reporting guideline for randomized trials [32]. However, there was no mention or link to that guideline on the website of the journal. By requiring the use of reporting guidelines and checklists by authors, editors and peer-reviewers can improve reporting of RCT methodology. This way the journals can reduce omission of crucial information, selective reporting, and presentation of data in a confusing and misleading way [33–35].

It is possible that some of the examined trials were organized well, but authors did not report the methodology properly. However, severely low scores we gave for all included trials suggests substantial problems with the methodology used in the national trials. One issue remaining from Soviet times is the absence of special courses for researchers in how to design and perform the trial. The lack of qualified specialists in biostatistics, who could contribute to a better organization of studies, is an additional factor lowering quality of the controlled trials. We found very low involvement of biostatisticians in the examined trials. Authors of reports find nothing special in reporting only desirable effects of interventions or in the concealing limitations of their study. Ignorance about or concealment of drug companies’ interests provides good ground for them to promote their drugs. It seems to become a common practice that drugs show their “positive” effects in post-Soviet countries, while having insufficient evidence for their effectiveness in the rest of the world.

### Limitations

We accept that conclusions about the quality of the published reports should be based on the critical consideration of our findings. The methodology and the tool used for assessing the reports may have internal limitations. The problem of varying degree of agreement among evaluators was described in the studies [15, 36, 37]. In our study, the cases of disagreement were found in the process of rating by three basic criteria. In that stage, the *κ*-index indicated moderate or substantial agreement between the evaluators. The role of consensus-based approach, used in the study, was important for this issue. The issue of blinding could also confound the results, in that both evaluators initially knew all characteristics of journals and published trials. However, an assessment of reliability did not prove the significant role of blinding on the Jadad’s quality score [14]. More detailed tools for evaluation, such as the Cochrane RoB or PEDro scales, are more comprehensive and their use could add benefits to the study. Our study is also limited in that it used non-random approach and included only reports published in a subset of national journals, and only those reported from 2007 to 2017. However, a historically similar organization of controlled trials by researchers as well as the same editorial policy of journals, in post-Soviet countries [1, 2], could suggest that our results can be applied not only to the most recently published trials but also to some other national journals.

## Conclusions

Despite the limitations of our study, we believe that quality of reports about controlled trials, in Uzbekistan, can be considered insufficient to contribute to the higher quality of care and patients’ safety. In the worst case, such condition can cause serious damage to the public health and lead to ineffective use of medical resources in the country. Therefore, the better reporting of RCTs and CCTs becomes a main goal of all stakeholders interested in the effective and safe healthcare in the country. It is clear that such strategy will require better training of editors and reviewers of journals, as well as potential researchers. We strongly recommend that national researchers carefully organize and report on their controlled trials, including procedures for the randomization of participants and the blinding of the interventions. We also encourage improving clinicians’ proficiency in English and their skills in searching for information and its critical evaluation. National journals should clarify their requirements for reporting of RCTs and CCTs, possibly using CONSORT as a checklist. A formal registration of trials, at least in the national level, could provide an additional benefit.

## Supplementary information

**Additional file 1: Supplementary file 1.**

## Data Availability

All data generated within this study are available in the Supplementary file 1.

## Abbreviations

CCT: Controlled Clinical Trial
CONSORT: CONsolidated Standards of Reporting Trials
ICMJE: International Committee of Medical Journal Editors
MoH: Ministry of Health
PEDro: Physiotherapy Evidence Database
RCT: Randomized Controlled Trial
RoB: Risk of Bias
WHO: World Health Organization
